# Creative use of the priority review voucher by public and not-for-profit actors delivers the first new FDA-approved treatment for river blindness in 20 years

**DOI:** 10.1371/journal.pntd.0006837

**Published:** 2018-11-15

**Authors:** Piero L. Olliaro, Annette C. Kuesel, Christine M. Halleux, Mark Sullivan, John C. Reeder

**Affiliations:** 1 UNICEF/UNDP/World Bank/WHO Special Programme for Research and Training in Tropical Diseases (TDR), World Health Organization (WHO), Geneva, Switzerland; 2 Centre for Tropical Medicine and Global Health, Nuffield Department of Medicine, University of Oxford, Oxford, United Kingdom; 3 Medicines Development for Global Health (MDGH), Melbourne, Australia; University of Florida, UNITED STATES

Neglected tropical diseases (NTDs) represent a significant disease burden, feeding a vicious cycle of permanent disability and poverty [1]. Better treatments and treatment access could break this cycle, but the significant financing requirements for development are a barrier, despite the public-health imperative. Traditional incentives for development and supply of new and/or innovative drugs, diagnostics, and vaccines—such as return on investment—do not apply, leaving philanthropy and government financing as the dominant mechanisms supporting new products for NTDs. The evidence of this “market failure” is damning: just 2% of the total research and development (R&D) spent is on diseases affecting 33% of the global population [2]. Time trends show no significant improvement, with a mere 1% of all new chemical entities approved between 1975 and 1999 [3] and between 2000 and 2011 [4] being for tropical diseases (NTDs and malaria) and tuberculosis. More diverse and sustainable mechanisms are required.

In 2007, the United States of America Congress created the priority review voucher (PRV) program [5] to encourage development of new products for certain “tropical diseases” (rare pediatric diseases and medical countermeasures were later added). If a “new drug application” (NDA) for an eligible indication is approved by the US Food and Drug Administration (FDA), the applicant is awarded a PRV. The PRV permits priority review of a subsequent NDA for a drug that the FDA would otherwise assign to standard review, thus reducing time-to-market by four to six months. This means gaining a competitive advantage, more sales, and greater profitability. The voucher can be redeemed by the same applicant or can be sold [6].

The impact of the PRV on the development of innovative and affordable medicines for “tropical diseases” and the intended public-health gains is questionable [7,8]. Here we show how the prospect of the voucher facilitated FDA approval of a new treatment for onchocerciasis, a milestone towards its availability to affected populations that otherwise might not have been achieved.

Onchocerciasis (river blindness) is one of the 22 PRV-eligible “tropical diseases.” It is caused by the parasitic worm *Onchocerca volvulus*, transmitted to humans by the blackflies *Simulium* spp. Around 185 million people are at risk of infection, [9] which leads to severe itching, disfiguring skin conditions, and visual impairment, including blindness. More than 99% of infected people live in 31 African countries [10]. Ivermectin, the drug on which current onchocerciasis control and elimination programs are based, is donated and distributed as preventive chemotherapy to entire populations living in transmission areas [11,12].

Screening and development programs for NTDs, initiated at the Special Programme for Research and Training in Tropical Diseases (TDR; hosted by the World Health Organization) in the 1970s, identified moxidectin as a candidate for clinical development [12]. TDR entered into arrangements with the pharmaceutical company owning moxidectin for co-development and registration for onchocerciasis. Moxidectin, like ivermectin, is a macrocyclic lactone with extensive history of veterinary use. In Phase II [13] and III [14] clinical trials, moxidectin proved superior to ivermectin in reducing skin levels of the parasite life stage (microfilariae), which causes disease symptoms and is taken up by blackflies resulting in transmission. Fewer people with skin microfilariae (and fewer skin microfilariae) for longer—compared with ivermectin—should not only reduce morbidity but also parasite transmission and thus time to onchocerciasis elimination.

Following withdrawal of the co-development partner in 2011, TDR sought another partner, without whom registration could not be achieved. Medicines Development for Global Health (MDGH) is a not-for-profit biopharmaceutical company founded in 2005 to tackle health inequity by developing and registering medicines for neglected diseases. The PRV program was pivotal to completing moxidectin’s development and registration: MDGH leveraged the PRV for a US$13 million investment by the Global Health Investment Fund (GHIF), making this the first social impact investment raised specifically on the potential value of a PRV. The funds were used to complete manufacturing and nonclinical and clinical development activities necessary for regulatory compliance as well as to write the regulatory dossier for submission to the FDA. In total, MDGH and TDR each contributed around US$15 million to repurpose moxidectin from animal to human health (relevant investment by TDR’s previous for-profit partner is unknown but very likely exceeds US$20 million).

The FDA approved moxidectin for onchocerciasis on 13 June 2018 and awarded MDGH a PRV [15]. Moxidectin fulfils several PRV objectives: the NDA was genuinely new because moxidectin had not been registered elsewhere and is the first onchocerciasis treatment approved by the FDA in 20 years (30 years after ivermectin registration in France in 1987). Furthermore, proceeds from the PRV sale will stay in the neglected disease sector, supporting access to moxidectin through subsidies and funding further R&D on moxidectin and other products for NTDs; finally, it brings new prospects for the onchocerciasis endgame.

The moxidectin story illustrates how partners motivated not by profit but by public good can use the PRV program, designed around the market forces motivating the for-profit sector, to meet its intended objectives. To do this, certain elements must converge: public funds and public-health priority setting, not-for-profit drug development and regulatory know-how, and commitment to invest PRV gains into furthering affordable access and additional R&D for NTDs.

Threats to the PRV scheme achieving its objectives are becoming increasingly obvious, not least of which is oversupply of PRVs [16]. So far, 23 vouchers have been issued, including 5 in 2018 to date. These numbers reflect the PRV extension to “rare pediatric diseases” and “medical countermeasures”: only 7 out of 23 PRVs were for “tropical diseases” indications. With increased numbers, the PRV market valuation has dropped from peak US$350 million in 2016, via an average US$130 million in 2017 to US$81 million for the last recorded sale in 2018 [6]. We concur with Ridley and Régnier [16] that a PRV value below US$100 million no longer incentivizes drug development for “tropical diseases”.

This is particularly the case for nonprofit organizations that depend on public or philanthropic funding and/or need to raise money from “investment” funds. Moxidectin is a case in point: even though repurposing is comparatively cheap, the estimated total cost to bring moxidectin to FDA registration may well have exceeded US$50 million. In contrast to public and/or philanthropic funders, investment funds inevitably require reimbursement and significant return on investment. This substantially reduces the proceeds from the PRV sale available for additional work required to bring the drug to the target countries, including registration, additional studies to inform guidelines and policy, and finally, to make it available and affordable to those who need it.

Another weakness of the PRV program is that companies granted a PRV have no obligation to make the FDA-approved product available at an affordable price. While MDGH is committed to subsidize procurement of moxidectin as needed, experience with other drugs for which a PRV was granted shows that without such obligations, the PRV program will not achieve its ultimate objective, i.e., improved treatment for NTDs.

The inherent weaknesses of the PRV program risk the viability of an initiative that we have shown can indeed make an important contribution to addressing market failure. Codifying access and pricing obligations is an essential change that would help preserve this important incentive program.
